# The Effect of Using a Client-Accessible Health Record on Perceived Quality of Care: Interview Study Among Parents and Adolescents

**DOI:** 10.2196/50092

**Published:** 2024-04-23

**Authors:** Janine Benjamins, Emely de Vet, Chloe A de Mortier, Annemien Haveman-Nies

**Affiliations:** 1 Chairgroup Consumption and Healthy Lifestyles Wageningen University & Research Wageningen Netherlands; 2 Icare JGZ Meppel Netherlands; 3 Stichting Jeugd Noord Veluwe Nunspeet Netherlands; 4 University Collega Tilburg Tilburg University Tilburg Netherlands; 5 Department of Health Services Research Care and Public Health Research Institute Faculty of Health Medicine and Life Sciences, Maastricht University Maastricht Netherlands; 6 School of Health Professions Education Faculty of Health Medicine and Life Sciences Maastricht University Maastricht Netherlands; 7 Knowledge Instiute of Medical Specialists Utrecht Netherlands; 8 GGD Noord-en Oost Gelderland Warnsveld Netherlands

**Keywords:** electronic health record, EHR, personal health record, child health services, child health, child welfare, adolescent health services, pediatrics, parent, care quality, perceived quality of care, patient experience, client perception, quality of care, parent, adolescent, patient portal, client portal, qualitative study

## Abstract

**Background:**

Patient-accessible electronic health records (PAEHRs) are assumed to enhance the quality of care, expressed in terms of safety, effectiveness, timeliness, person centeredness, efficiency, and equity. However, research on the impact of PAEHRs on the perceived quality of care among parents, children, and adolescents is largely lacking. In the Netherlands, a PAEHR (Iuvenelis) was developed for preventive child health care and youth care. Parents and adolescents had access to its full content, could manage appointments, ask questions, and comment on written reports.

**Objective:**

This study aims to assess whether and how using this PAEHR contributes to perceived quality of care from a client’s perspective.

**Methods:**

We chose a qualitative design with a phenomenological approach to explore how parents and adolescents perceived the impact of using a PAEHR on quality of care. In-depth interviews that simultaneously included 1 to 3 people were conducted in 2021. In total, 20 participants were included in the study, representing parents and adolescents, both sexes, different educational levels, different native countries, and all participating municipalities. Within this group, 7 of 13 (54%) parents had not previously been informed about the existence of a client portal. Their expectations of using the client portal, in relation to quality of care, were discussed after a demonstration of the portal.

**Results:**

Parents and adolescents perceived that using Iuvenelis contributed to the quality of care because they felt better informed and more involved in the care process than before the introduction of Iuvenelis. Moreover, they experienced more control over their health data, faster and simpler access to their health information, and found it easier to manage appointments or ask questions at their convenience. Parents from a migratory background, among whom 6 of 7 (86%) had not previously been informed about the portal, expected that portal access would enhance their understanding of and control over their care processes. The parents expressed concerns about equity because parents from a migratory background might have less access to the service. Nevertheless, portal usability was regarded as high. Furthermore, both parents and adolescents saw room for improvement in the broader interdisciplinary use of Iuvenelis and the quality of reporting.

**Conclusions:**

Using Iuvenelis can contribute to the client-experienced quality of care, more specifically to perceived person centeredness, timeliness, safety, efficiency, and integration of care. However, some quality aspects, such as equity, still need addressing. In general, client information about the portal needs to be improved, specifically focusing on people in vulnerable circumstances, such as those from migratory backgrounds. In addition, to maximize the potential benefit of using Iuvenelis, stimulating a person-centered attitude among professionals is important. Considering the small number of adolescent participants (n=7), adding quantitative data from a structured survey could strengthen the available evidence.

## Introduction

### Background

In the implementation and optimization of health care services, assessing the quality of care is an important topic. Quality of care is a broad concept, and it encompasses various aspects of health care. Most commonly used is the Institute of Medicine’s definition of quality of care, which distinguishes 6 different domains: safety, effectiveness, timeliness, patient centeredness, efficiency, and equity [1]. Patient safety refers to the notion that provided care should prevent patients from harm [1]. Effectiveness reflects the use of appropriate interventions and treatments [1]. Timeliness refers to delivering health care services on time [1]. Patient centeredness is about tailoring care to the unique patient’s needs and preferences and engaging them and their proxies in decision-making [1,2]. Efficiency deals with how well resources are used and about avoiding waste [1]. Equity ensures everyone has equal access to the best possible care, independent of personal characteristics or geographic location [1]. Traditionally, quality of care has been approached from a professional’s perspective, aiming to increase the likelihood of desired health outcomes. In 2015, the World Health Organization (WHO) reformulated the term patient centeredness into person centeredness, emphasizing that patients are more than just their health condition and proposing a broadened scope for health and well-being [3]. With this pivot shift from conventional biomedical health care models to a more holistic approach, patient experiences have become an important health care quality outcome, and patient-reported experiences have evolved into important indicators for quality of care [4,5].

Patient-accessible electronic health records (PAEHRs) are assumed to enhance the quality of care because they provide users with information about their health and health care [6-8]. Information can be provided in a one-way manner, by sharing health data in a patient portal or interactively when the system supports messaging between patient and care provider [9-12]. Either way, providing patients with their health data promotes empowerment and enhances people’s engagement in their care plans [6,7,13]. Consequently, health consciousness (ie, the inclination to take health actions), therapy adherence, and self-management of health improve, all of which contribute to better health outcomes [8,9,13-16]. Moreover, transparency of PAEHRs is reported to enhance patient safety, for instance, because patients can identify errors in their health records and have them corrected [12,17,18].

### PAEHRs in Adolescent Health Care

The growing body of literature reporting the effect of using PAEHRs on quality of care predominantly stems from adult health care. Research on the impact of using PAEHRs on the quality of care among children, adolescents, and their parents is limited because the development of PAEHRs for these target groups is delayed by age-specific challenges regarding autonomy and confidentiality [19,20]. Meeting these challenges during the development of PAEHRs is important because research shows that adolescents only share information with professionals who assure their confidentiality [21-23].

The protection of confidentiality and access to health information differs depending on the country or state. While there are different legal measures in place to safeguard confidentiality, all health care systems face the challenge of transferring access rights from parents to adolescents [20,24,25]. Initially, parents have the right to their child’s health information, but as children grow into adolescence, and therefore in capacity and autonomy, these rights are transferred to the adolescent [26,27]. This transfer, varying across and within countries, can be gradual, with both parents and adolescents having access, or occur at a specific age [20,24,25]. Solutions for the emerging autonomy and confidentiality issues aim to balance adolescent autonomy and confidentiality with parental involvement [26-28]. In the United States, laws explicitly safeguard parents’ rights to access their children’s health information [25,29]. Contrastingly, countries such as Canada, the United Kingdom, the Netherlands, and most Scandinavian nations more strongly emphasize the rights of adolescents, depending on their capacity and maturity [24,30]. Restrictions on access to health information for both parents and adolescents vary globally, from shared access during a specified period to no access at all during adolescence [20,24]. The age at which adolescents can access their health information differs from any age in Finland and Estonia to 18 years in Austria and New Zealand [20,24]. Consent from either the adolescent or the parent may be necessary, with certain jurisdictions permitting adolescents to restrict parental access [20,25].

### Objectives

In the Netherlands, a PAEHR named Iuvenelis has been developed for children, adolescents, and their parents. Iuvenelis is used in an interdisciplinary manner in preventive child health care and youth care. It is accessible to adolescents aged ≥12 years and to parents of children aged from 0 to 16 years. Investigating the impact of using Iuvenelis on perceived quality of care among adolescents and parents will contribute to knowledge about using PAEHRs in an age group that is evolving toward autonomous adulthood. This study aimed to investigate how Dutch parents and adolescents visiting preventive health care and youth care perceived the impact of using a client-accessible interdisciplinary health record on quality of care, exploring both the experiences of active users and the expectations or first impressions of nonusers.

## Methods

### Research Design

A qualitative design with a phenomenological approach was chosen to explore how parents and adolescents perceived the impact of using Iuvenelis on the quality of care [31]. A total of 12 in-depth interviews with 1 to 3 people simultaneously were conducted between October 11 and November 25, 2021. We reported our qualitative study according to the COREQ (Consolidated Criteria for Reporting Qualitative Studies) [32]. Multimedia Appendix 1 contains the completed COREQ checklist for this study.

### Study Setting

The Dutch North Veluwe region consists of 6 municipalities. These municipalities commissioned 2 organizations providing preventive child health care to children aged 0 to 3 years and children aged 4 to 18 years and 1 organization providing youth care to integrate their services in the Centre for Youth and Family (CJG). The CJG is a network organization that houses professionals from the 3 parent organizations involved. Since 2015, the CJG has provided preventive health care to all 38,000 children aged from 0 to 18 years in the region and provided additional youth care for children and families with behavioral or sociopsychological problems [33]. Both preventive child health care and youth care refer to parents, children, and adolescents as clients rather than as patients. Using a participatory approach, the CJG in 2016 developed a quality standard for their services, following the European “Quality 4 Children” protocol [34]. In dialogue sessions with parents and adolescents, they jointly wrote a document that defined quality of care from a client’s perspective [35]. The document establishes 3 core values for quality—“child-centredness,” “partnership between family and professionals,” and “families in charge when decisions are made”—and describes the corresponding supportive professional behavior for each value [35]. Supporting the integration of services, the electronic health record “Iuvenelis” was built, to which all CJG professionals report. Furthermore, to support client autonomy and collaboration between professionals and families, Iuvenelis includes a tethered client portal in which parents and adolescents can read everything professionals report, such as visit notes, measurements, test results, and referrals. They can manage appointments, send secure messages to professionals, ask questions, comment on written reports, and request corrections of errors. Compliant with Dutch legislation, adolescents receive automatic access to the portal at the age of 12 years [36]. At the same moment, the portal closes for parents, who have a legal right to access Iuvenelis until their child is 16 years of age. However, this right can only be effectuated when their child personally grants permission. When parents are granted access to their child’s record between 12 and 16 years of age, their child can still have single visit reports shielded from them. Iuvenelis was introduced in September 2019.

### Study Population and Inclusion

The study included the parents of children aged 0 to 16 years and adolescents aged ≥12 years, living in the North Veluwe region, further referred to as clients. Clients who visited the CJG in September 2021 were invited personally by CJG professionals, and some general characteristics were reported, such as sex, age, educational level, and native country. Clients who expressed interest in participating were contacted by email or phone to explain the nature and purpose of the interview and to make an appointment. Where feasible, clients were invited to join focus group interviews at a CJG location. Those unable to attend a group session were offered an individual or dual interview live at the location of their choice or on the web. Purposive sampling ensured a varied group representing both sexes, parents and adolescents, various educational levels, active users of Iuvenelis and nonusers, both visitors of preventive health care and youth care, and inhabitants from all participating municipalities. We included parents from native Dutch and migratory backgrounds. In this paper, we use the term migratory background for immigrants who moved to the Netherlands, regardless of their command of the Dutch language. In total, 12 interviews were conducted with 20 participants. Apart from 7 (58%) individual interviews, 2 (17%) double and 3 (25%) triple interviews were conducted. Except for 1 (8%) triple interview with a mother and her 2 teenage children, group interviews consisted of only parents or only adolescents, and respondents did not know each other.

### Data Collection

To create an interview topic guide (Multimedia Appendix 2), a working session was convened with an interdisciplinary expert panel of 8 professionals. On the basis of the CJG quality standard and the overarching Institute of Medicine framework [1], they explored what aspects of client-perceived quality of care could be influenced by using Iuvenelis. Textbox 1 presents the main topics from the semistructured interview guide.

**Client interview main topics, with sample questions from the interview guide.**

**General**

- Are participants acquainted with Iuvenelis?
- How have their experiences been in general?
- If they were not acquainted, what are their first impressions?

**Safety**

- How do participants feel about security of their data?
- How do participants feel about detecting errors?
- How do participants value the view log?

**Effectiveness**

- How do participants experience completeness and understandability of reports in Iuvenelis?
- How do participants value professional expertise?

**Timeliness**

- How do participants experience the possibility of 24/7 access to their health data?
- How do participants experience the possibility to manage their own appointments?
- How do participants experience the possibility to ask questions at their convenience?

**Person centeredness**

- To what extent do participants perceive an influence of using Iuvenelis on client-professional collaboration or communication?
- To what extent do participants perceive an influence of using Iuvenelis on equal relationship?
- To what extent do participants perceive an influence of using Iuvenelis on sense of ownership?

**Efficiency**

- How do participants experience collaboration between disciplines through Iuvenelis?
- How do participants experience the use of interdisciplinary shared care plans?

**Equity**

- How do participants experience ease of access and ease of use?
- How do participants experience comprehensibility of record content?
- Were participants informed about the existence of Iuvenelis?

All participants were interviewed once by an experienced female interviewer (JB). For the first 6 of the 12 (50%) interviews, a female research assistant (CAdM) assisted as an observer and note-taker. Individual interviews lasted 30 to 60 minutes, and double and triple interviews lasted 90 minutes. When the participants were not acquainted with the client portal, the first part of the interview was used to demonstrate its functionalities in real time, followed by the main interview, which then focused on expectations and first impressions instead of experiences. Every interview was audio recorded, supplemented by note-taking, and by video recorded for web-based interviews.

### Data Analysis

The interviewers transcribed all interviews verbatim for analysis. A member check was conducted with all participants to affirm transcript accuracy. Data were analyzed in ATLAS.ti (version 9; ATLAS.ti Scientific Software Development GmbH). On the basis of the topic list with the 6 domains of quality of care as a framework, a preliminary codebook was written. In accordance with best practices, data collection and analysis were conducted in an iterative, cyclical process, checking for data saturation. The interviewing authors (JB and CAdM) conducted a thematic analysis, rereading and coding all transcripts independently [37,38]. After coding a full transcript, the 2 researchers discussed discrepancies in coding until consensus was reached. Simultaneously, in a continuous process, additional codes were added to the codebook, coding definitions were refined, and transcripts were recoded when necessary. Saturation was discussed during analysis and was reached after 12 interviews. Subsequently, JB and CAdM grouped all codes into major themes and discussed the interpretation of themes with all authors.

### Research Team and Reflexivity

The interviews were conducted by a researcher working as a policy advisor at the CJG and a research assistant, both trained in qualitative research. Although 1 interviewer worked in the CJG, no working relationship had been established with any of the participants before the study. Every interview started with an introduction of the interviewers and an explanation of the study goal. Combining an experienced researcher with inside knowledge of the CJG and Iuvenelis (JB) with a young researcher from outside the CJG (CAdM) had 2 advantages: first, when present during the interviews with adolescents, the younger researcher could identify easily with the participants and vice versa; second, during analysis, comparing observations and discussing interpretations from both inside and outside perspectives enriched the process of interpretation and limited the risk of bias.

### Ethical Considerations

The study was carried out following relevant guidelines and regulations, complying with the Netherlands Code of Conduct for Scientific Practice. On these grounds, the research protocol was approved by the Social Sciences Ethics Committee of Wageningen University (2018-24-Benjamins). All participants received an invitation beforehand with information about the study and gave explicit verbal consent at the beginning of the interview. Each interview was recorded and transcribed verbatim, including verbal consent.

## Results

### General Characteristics

Of the 20 participants, 13 (65%) parents and 7 (35%) adolescents were interviewed individually (n=7, 35%), in pairs (n=4, 20%) or in triplets (n=9, 45%). Initially, 23 participants were included, of whom 3 (13%) dropped out due to agenda mismatches. The participants represented both sexes, parents, and adolescents from different educational levels, from native Dutch and migratory backgrounds, and from all involved municipalities and also represented those making use of preventive child health care and youth care services. All adolescents were making use of youth care services. (Table 1).

A total of 35% (7/20) of the participants were not acquainted with the client portal before the interview, and 85% (6/7) of them were from a migratory background. Of the participants who were acquainted with the client portal, 46% (6/13) had received information from a CJG professional and, 54% (7/13) had discovered the portal through a questionnaire about Iuvenelis. In total, 30% (6/20) of the participants came to the CJG office, 50% (10/20) of them were interviewed in their own homes, and 20% (4/20) of the participants had web-based interviews.

**Table 1:** Characteristics of participants in the client interviews.

		Parents (n=13), n (%)	Adolescents (n=7), n (%)	Total (N=20), n (%)
**Sex**				
	Male	2 (15)	2 (29)	4 (20)
	Female	11 (85)	5 (71)	16 (80)
**Educational level**				
	High	3 (23)	2 (29)	5 (25)
	Middle	4 (31)	3 (43)	7 (35)
	Low	6 (46)	2 (29)	8 (40)
**Native country**				
	Netherlands	6 (46)	7 (100)	13 (65)
	Kosovo	1 (8)	0 (0)	1 (5)
	Syria	1 (8)	0 (0)	1 (5)
	Afghanistan	2 (15)	0 (0)	2 (10)
	Thailand	2 (15)	0 (0)	2 (10)
	Sudan	1 (8)	0 (0)	1 (5)
**Municipality**				
	Oldebroek	1 (8)	1 (14)	2 (10)
	Elburg	1 (8)	0 (0)	1 (5)
	Nunspeet	2 (15)	1 (14)	3 (15)
	Harderwijk	5 (38)	3 (43)	8 (40)
	Ermelo	3 (23)	2 (29)	5 (25)
	Putten	1 (8)	0 (0)	1 (5)
**Visiting preventive child health care or youth care**				
	Preventive child health care	10 (77)	0 (0)	10 (50)
	Youth care	3 (23)	7 (100)	10 (50)
**Acquainted with the portal**				
	Yes	6 (46)	7 (100)	13 (65)
	No	7 (54)	0 (0)	7 (35)

### Interview Outcomes

A code tree (Multimedia Appendix 3) was created with branches for all 6 aspects of quality of care: safety, effectiveness, timeliness, person centeredness, efficiency, and equity [1]. One additional theme emerged, related to professional attitude and behavior. Because this theme is linked with person centeredness, we divided the theme of person centeredness into 2 subthemes: client perspective and professional attitude. Most expressions from the participants could be coded in the domain of person centeredness (668/1749, 38.19%), followed by safety (382/1749, 21.84%), equity (337/1749, 19.27%), timeliness (158/1749, 9.03%), and efficiency (135/1749, 7.72%), whereas effectiveness was mentioned the least (69/1749, 3.95%). When experiences across quality-of-care domains were compared, it appeared that positive experiences were expressed for person centeredness, safety, and timeliness, whereas the domains equity and effectiveness evoked predominantly expressions of concerns. The participants expressed mixed feelings about the domain efficiency. In the following paragraph, more in-depth analyses of the participants’ reflections on individual dimensions of quality of care will be presented, starting with the domain that generated the highest number of codes.

### Person Centeredness

#### Subtheme A: Client Perspective

Both parents and adolescents reported that rereading information in the client portal contributed to person centeredness because it helped them to *recollect* what had been discussed during a visit, to *get an overview* over a longer period, and to *prepare* for the next visit:

Sometimes it is so crowded in my head. Then I start thinking: what was it all about?

Mother, 2 children, respondent 7.2

It’s more like when I am struggling with something that we have discussed earlier that I think: Hey, wait a minute. Didn’t we already talk about this once? And I can reread our conversation.

Female adolescent, aged 17 years, respondent 10

Using the client portal to get an overview was even more important for the parents with a migratory background, although only 1 of them had been using the portal before the interview. However, after watching the portal demonstration and accessing their own child’s health record, all parents from a migratory background considered access to the client portal to be very valuable. They expected that both rereading and reading with others would be vital. Rereading, and using a web-based translation tool when they did not comprehend the Dutch text, would help them to get a *better understanding* of what was discussed during a previous visit. A total of 50% (3/6) of the mothers with a migratory background had partners who understood Dutch better than they did. Rereading together after a mother’s visit to the CJG would *provide* the father with all relevant *information* and would help the mother *recollect* what was discussed or provide her with information that she had not grasped yet during the visit:

This one (client portal), this is good! My husband always asks: “How big was his head, how tall was he and how many kilos.” And then I go: “Oh my goodness, I forgot! Do I need to memorize that?” Now I can say: “Hey, you can log in and see for yourself what has happened.”

Mother, 1 child, respondent 2.1

*Involving relatives* in one’s care was an aspect of person centeredness that not only the parents with a migratory background reported as a benefit from access to the client portal. Most parents valued that a partner who had not been present at the physician’s visit could read the notes afterward. For adolescents, it felt easier to have parents read a visit report than to recall the whole conversation themselves, although they also valued the possibility of actively withholding information from their parents if they wanted to. Finally, rereading with relatives or friends was reported as helpful as well, when *preparing* for a next visit, or when *decisions* had to be made about the care process:

I have a Syrian friend who does not speak Dutch. Her daughter has a growth problem. I helped her and we took the information from the growth chart in this portal, bringing it with us to the hospital.

Mother, 3 children, respondent 11

Being able to reread information, the parents and the adolescents felt *well informed* and *engaged* in their care plan. They also valued being part of the reporting process, discussing beforehand what should be reported and how. The combination of reporting together and rereading information enhanced their sense of *ownership* and contributed to *equal client-professional collaboration*:

Now I know, because I can check myself, when my children need vaccinations

Father, 5 children, respondent 3.2

You construct the report together, so to speak, and you can both navigate the plan a little.

Mother, 2 children, respondent 7.3

Both parents and adolescents would like to have *more ownership* than was facilitated by the client portal. Some parents expressed the need to *add* more *information* to Iuvenelis to create a full overview of all health and welfare issues concerning their child. Adolescents wanted to be more in control of who accessed their health records; they wanted to *actively give access* to professionals or at least be able to see beforehand who had access to their record instead of reading afterward in their view log who had accessed their health information:

At least I want to see beforehand which professional is authorized to access my health record, instead of seeing who has accessed my record afterwards.

Male adolescent, aged 17 years, respondent 5.1

#### Subtheme B: Professional Attitude

Numerous participants emphasized that a *professional attitude* was an important underlying condition to deliver person-centered care and to experience the possible benefits of using Iuvenelis. The transparency of Iuvenelis contributed to a sense of *trust*, but only if professionals reported respectfully, showing that they did take clients seriously. Being able to see in a view log who accessed your health record was considered reassuring and enhanced trust. A mother stated the following:

You should consider very carefully how you report, because you are inviting me: “Go ahead, read it.” You are giving full access to the health record.

Mother, 2 children, respondent 7.3

On the other hand, trust could be damaged if professionals did not report respectfully or did not respect a client’s privacy. After experiencing numerous instances where professionals were speaking about her, 1 parent chose not to access the client portal, to protect herself from losing trust in her current care provider:

I have decided that I trust “X” completely. Why should I read my health record when I do not need to and take the risk to read something that might harm that trust?

Mother, 2 children, Respondent 9

### Safety

Both parents and adolescents were satisfied with the *security* of their *health data* and the way professional authorization was organized. They generally valued the possibility to see in their view log who accessed their health record. Adolescents all valued their *right to decide* about access for their parents. Knowing how safety was warranted was an important factor contributing to their *trust* in the system:

This afternoon I saw that someone had accessed my daughter’s record. But I remembered I approved that person. It’s nice to know that my approval is needed beforehand.

Mother, 4 children, respondent 7.1

I had problems with my parents, and I don’t know if that’s still in all those documents. Then it is nice indeed that you can decide, what they can and can’t see.

Male adolescent, 17 years, respondent 5.1

However, half of the portal-using participants were well informed about the privacy and data security measures, and knew where to find the view log. For 1 adolescent, the view log was a reminder that professionals were discussing her situation without her being present, which she did not appreciate:

Although I like seeing who has accessed my health information, it also gives me stress. Because once they discussed my condition in a meeting with several people and I was not there. They were talking about me without me, so to speak, and that’s not okay. When I check the view log that situation comes back in mind.

Female adolescent, aged 18 years, respondent 5.2

Can other people [outside the CJG] see my child’s record? How do I know that you don’t give it to other people? Because everything is web-based.

Mother, 1 child, respondent 2.1

*Correcting errors* is generally considered a part of the element “safety” [12]. Throughout the interviews, 2 adolescents and 3 parents encountered registration errors or missed appointments without follow-up when checking their portal. They said identifying errors did not upset them. Quite the reverse; they appreciated the possibility to detect errors, report them, and have them corrected. Moreover, being able to correct mistakes increased their sense of ownership over their care process. The parents said it was important to correct found errors, whereas adolescents said they would not ask for correction:

Sometimes things go wrong. For example, E had missed a vaccination. So now we can check the record ourselves and see which vaccination he needs.

Father, 5 children, respondent 3.2

### Equity

Independent of their native country and educational level, participants thought very positively of the client portal’s *usability*. The portal was experienced as easy-to-use and intuitive. The parents and the adolescents could log on to the system easily using digital ID, because people had familiarized themselves with this verification procedure during the COVID-19 pandemic. Usability on mobile phones was also considered good:

Logging in with DigiD makes things easier actually, solving the whole hassle of passwords.

Mother, 4 children, respondent 7.1

For me, it must be well-organised and then it’s good. The way it is constructed right now, it’s clear, uncluttered and you can read everything. I think I will look more often.

Mother, 2 children, respondent 7.3

The parents and the adolescents also considered most recorded content *comprehensible*. However, some portal features, for example, vaccination overview and planning appointments, required explanation, and the parents and the adolescents sometimes encountered jargon or incomprehensible abbreviations:

I understood most things I read. But I thought about some information from when I was a little kid, some expressions: that must be only for doctors.

Female adolescent, aged 18 years, respondent 12

The most serious concern expressed by parents was that not all clients were informed *equally* about the existence of Iuvenelis. A total of 7 (35%) out of 20 participants had not received any information about Iuvenelis before the interview, and 86% (6/7) of them were from a migratory background. One parent from a migratory background did use the client portal to manage appointments but was not aware that she could also reread visit reports:

If I had not been here, I would not have known anything about it at all, and that’s a shame.

Mother, 1 child, respondent 2.3

The parents presented many options for improving communication. Emphasizing the importance of providing more equal information to all population groups, 1 parent offered to participate in information meetings with mothers from migratory backgrounds:

Some mothers (with a migratory background) are unsure about their language proficiency. For them, it is easier to do it through the internet.

Mother, 3 children, respondent 11

### Timeliness

The client portal’s 24/7 accessibility did not contribute to *faster* access to care. However, it did provide parents and adolescents with the opportunity to ask questions or schedule appointments easily and at their *convenience*. Especially, parents valued this opportunity as *time saving*, including the immediate access to their health information without the interference of a CJG professional:

Suppose I get very anxious during the weekend about certain behaviour I observed. I would prefer to search for information right then and there, instead of sending an email and waiting several days until someone responds. I think it’s a plus that I can check the client portal and ask my questions immediately.

Mother, 2 children, Respondent 7.3

I rescheduled my appointment once through the portal. Very convenient and timesaving!

Mother, 2 children, respondent 7.2

### Efficiency

In Iuvenelis, all CJG professionals had access to all relevant information stored in the same place, which was considered an advantage contributing to efficiency. Consequently, the parents and the adolescents did not have to repeat their stories when visiting a new professional in the CJG:

I think it is very convenient when you visit several people in the same period that all information is in one place. So, they can make use of each other’s information.

Female adolescent, 15 years, respondent 6

However, both parents and adolescents saw room for improvement in expanding Iuvenelis toward other care providers and in a more active role for themselves in uploading information from other care providers in their client portal. They felt that if all their health data were stored in one place and accessible to all their care providers, it would be easier for both care providers and clients themselves to create a clear overview and manage their care:

I hope lines between all professionals will be shorter. Eventually, I hope my children will have all their health data in this record, that this will be their complete and only health record.

Mother, 2 children, respondent 8.3

### Effectiveness

Parents and adolescents did not associate using Iuvenelis with effectiveness. Although a fully accessible health record allows clients to engage in the management of their care process, none of the participants commented on the actual care process and whether the right choices had been made.

Parents and adolescents did comment on the process and quality of reporting: they felt that reporting quality could be improved. Some reports contained mistakes, and some were incomplete or missing. One parent expressed the concern that reports were sometimes prejudiced, elaborating on risk factors and neglecting protective factors:

They only report what is wrong. Do you know what could really help? If you would read in your child’s record what is going well if someone would write down what a lovely little boy he is.

Mother, 2 children, respondent 9

## Discussion

### Principal Findings

With this study, we explored how parents and adolescents visiting preventive health care and social care perceived the quality of care when using Iuvenelis. Both the experiences of active users and the expectations or first impressions of nonusers were included. The results suggest that using Iuvenelis contributed to some, but not all, aspects of quality of care. On the positive side, parents and adolescents felt better informed and expressed more engagement in the care process than before introduction of Iuvenelis. They felt more in control of their health data, reported having faster and simpler access to their health information, and found it easier to manage appointments or ask questions at their convenience. Portal usability and data safety were regarded as high, and interdisciplinary collaboration in Iuvenelis was considered to enhance efficiency. The parents from a migratory background expected that portal access would give them a better understanding of and more control over their care processes.

However, parents expressed concerns about possible unequal access due to a lack of information for the parents from a migratory background. Furthermore, both parents and adolescents saw room for improvement in the broader interdisciplinary use of Iuvenelis. Finally, they felt that effectiveness could be improved by more complete reporting regarding protective factors as well as risk factors.

### Comparison With Prior Work

#### Overall Contribution to Quality of Care

Previous research investigating quality of care in relation to using PAEHRs predominantly focused on adult health care. These studies reported largely the same outcomes as our study, although described from a care provider’s perspective. Using a PAEHR was reported to contribute to person centeredness [7,39,40], safety, and efficiency [16,39,40]. Contrary to this study, prior studies also show a positive impact of using a PAEHR on effectiveness [16,39,40]. Some studies report that patient portals enhance timeliness through messaging functionalities or quicker access to results [41-45].

#### Person Centeredness and Professional Perspective

Some participants emphasized the importance of a person-centered professional attitude, which they considered fundamental for Iuvenelis’ contribution to quality of care. When professionals reported respectfully in Iuvenelis, this enhanced the client’s trust in their care providers, whereas earlier experiences with professionals not respecting a client’s privacy damaged that trust. An extensive review by Scholl et al [46] generated a patient-centered care model that places a professional’s attitude central in the delivery of person-centered care. In this model, delivering patient-centered care relies on professionals embracing a person-centered attitude characterized by respecting a patient’s unique preferences and needs, building a professional-patient relationship based on equality, and viewing a patient’s health from a biopsychosocial perspective [46]. Leeuwis and Aarts [47] stated that complex interventions, such as technological innovations, usually require change on different levels. These changes, on a technological, organizational, and professional level, are considered interdependent [47]. In this case, implementing a PAEHR to enhance person centeredness is not only about introducing the technological tool; the implementation needs to address professional attitude and behavior as well. In turn, changes in professional behavior and attitude require adjustments at the organizational or institutional level. These interdependencies should be anticipated when organizations start implementing a PAEHR, and the necessary changes on an organizational and professional level should be planned and facilitated in addition to the development and implementation of the tool itself.

#### Equity

Equity emerged in this study as an issue of concern because most participants with a migratory background appeared to be unaware of the existence of a client portal, as opposed to 1 participant with a native background. Diving a bit deeper into this, anecdotal evidence may suggest that professionals hesitated to inform clients about the existence of the client portal when they noticed that a client’s knowledge of Dutch was limited. Unawareness of the existence of a patient portal has been reported as a main barrier for using a patient portal [48,49] and could be resolved by provider encouragement, which is an important contributor to portal use [50-52]. However, when providers selectively encourage certain groups of people to use a patient portal and neglect others, they could enhance disparity. Previous research shows that persons living in vulnerable circumstances, such as lower-educated people or persons from a migratory background, make less use of patient portals than average [17,42,53-58]. The literature on the digital divide reports that social exclusion can lead to digital exclusion and that the introduction of new technology then might unintentionally reinforce already existing health disparities [59-61]. In total, 2 studies investigating a provider’s role in patient portal use reported that professionals play a role in this reinforcement: higher-educated and White patients were more likely to report being encouraged by health care providers to use a client portal than lower-educated patients and patients from migratory backgrounds [50,51]. Antonio et al [62] stated in a review that “healthcare providers’ prejudgments may further exclude populations that are already underserved.”

This is an important issue to address because research shows that people, especially those living in vulnerable circumstances, experience benefits from using a PAEHR [42,43,63,64]. In our study, parents from migratory backgrounds reported that rereading their health information and sharing it with family members or friends would provide them with a better understanding of the care process and would increase their engagement in care. We concluded that ensuring that all clients are equally informed about the existence of a client portal is not only necessary to prevent further disparities but could even diminish existing disparities [65]. This may require adapted measures for specific population groups, for example, using informal meetings with the parents from migrant backgrounds to inform them in their language about Iuvenelis. In addition, professionals need to be made aware of the risk of the digital divide and of their crucial role in conquering this phenomenon.

#### Confidentiality

On the basis of the known bottlenecks to developing PAEHRs for adolescents [26,27], we expected data safety, confidentiality, and privacy to be an issue of concern for at least some of our participants. However, surprisingly, participants did not express concerns about their data safety. Adolescents did value highly how their confidentiality was protected and reported that this contributed to their trust in their care provider. Comparably, recent studies investigating adolescent use of PAEHRs suggest that adolescents are not concerned about their confidentiality when using a PAEHR [8,13,18,23]. A recent review investigated the experiences of parents and adolescents using a PAEHR in hospital, primary, and mental health care settings versus the expectations of parents and adolescents without access to a PAEHR. In this review, the authors found that parents and adolescents without access to a PAEHR anticipated confidentiality issues when using a PAEHR, whereas parents and adolescents using a PAEHR did not experience these issues [66]. In a similar vein, research that compared professionals’ general concerns about using PAEHRs beforehand with experiences after a period of using a PAEHR shows that anticipated worries were not always justified. For example, an expected increase in workload and excessively anxious patients did not occur after introducing PAEHRs [67-69]. Confidentiality issues could have been one of the expected problems that did not evolve. Another explanation of the contrast between expected bottlenecks and real experiences may be that the explicit focus in the literature on confidentiality issues has initiated specific awareness for this topic during the development of Iuvenelis and has led to the implementation of successful solutions.

#### Integrated Care

The participants considered the interdisciplinary use of Iuvenelis a contribution to efficiency and even expressed a need to expand the use of Iuvenelis to other disciplines outside the CJG. This would allow them to view all their health data in one place. Parents and adolescents stated that, in their opinion, this would contribute to efficiency. However, with their remarks, participants draw upon an additional aspect of quality of care, integrated care, that the WHO has added recently [1,70]. The WHO defines integrated care as “providing care that is coordinated across levels and providers and makes available the full range of health services throughout the life course.” The parents and the adolescents even challenged the CJG organizations to extend opportunities for interdisciplinary collaboration within Iuvenelis, facilitating them to gather all their health information here. With that challenge, the parents and the adolescents confirmed the value of the Dutch aim for integrated care in child health care and youth care [71]. This aim is also reflected in the recently established Healthy and Active Living Agreement between the Dutch government, municipalities, and public health associations [72], although it is not yet common practice throughout the country.

#### Differences Between Parents’ and Adolescents’ Experiences

Although parents’ and adolescents’ perceptions were similar in many aspects, differences were reported as well. Parents considered it more important to correct errors than adolescents and valued the web-based option to ask questions and manage appointments more highly than adolescents. Comparably, recent studies among adolescent patients show that adolescents are less likely to speak up about mistakes in their records than their parents [73,74], and are more reluctant to send direct messages in the PAEHR to their caregivers than adults [13,23]. Both parents and adolescents liked to share record content with their close ones, but adolescents also valued the opportunity to shield specific content from their parents when needed. Adolescents considered deciding who had access to their health information vital to exercising ownership over their health information. In line with this, a recent review reports that teens believe they should have control over what remains confidential in their medical records and what their parents can access through proxy portal accounts [23].

### Strengths and Limitations

Recruiting a well-balanced group of participants in this qualitative study was a strength of this study, compared to our previous studies on Iuvenelis, where adolescents were represented in small numbers and participants with migratory backgrounds could not be included [75,76]. The inclusion of the most important characteristics in this study enabled us to explore different client perspectives. Choosing a qualitative research design made it possible to collect rich, in-depth information about the client’s expectations of and actual experiences with using Iuvenelis.

Due to the COVID-19 pandemic, organizing focus groups proved to be difficult. Although some triple interviews could be organized, most participants were interviewed individually or in couples. Consequently, our study lacked some of the interaction that is usually generated in larger groups, which could be considered a limitation [77]. We partly managed to overcome this limitation because we collected and analyzed data in a continuously iterative process. This meant that topics that were brought up in the first interview could be explored further in the following interviews.

As JB had a role as a policy advisor in the CJG, she was able to introduce the participants to Iuvenelis who were not yet acquainted with the client portal, which allowed us to include more parents with a migratory background and to add valuable information to our data. However, combining a portal demonstration with an interview about how clients perceived the quality of care using this portal might have created a respondent bias: the interviewer’s positive attitude toward the client portal could have evoked socially desirable answers. To enhance trustworthiness, the interviewers followed the interview guide as closely as possible, allowing some adaptation to the conversational flow. A member check was conducted, transcripts were coanalyzed with a researcher with no connections with Iuvenelis or the CJG, and reporting followed the COREQ checklist [32,78].

### Conclusions

Using Iuvenelis is expected to contribute to experienced quality of care from the perspectives of both parents and adolescents, specifically to the aspects of person centeredness, timeliness, and safety. Parents and adolescents feel better informed, experience a greater sense of ownership, and are satisfied with data security and portal usability. Clients also report that using Iuvenelis contributes to integrated care. Some quality aspects, however, such as equity in portal access, still need addressing. In general, client information about the portal needs to be improved, specifically focusing on people in vulnerable circumstances, such as those from migratory backgrounds. In addition, to maximize the potential benefit of using Iuvenelis, stimulating a person-centered attitude among professionals is important. With our study, we have investigated parents’ and adolescents’ perspectives regarding all domains of quality of care. However, considering the small number of adolescent participants, adding quantitative data from a structured survey could strengthen the available evidence.

## Appendix

Multimedia Appendix 1 Completed COREQ (Consolidated Criteria for Reporting Qualitative Studies) checklist.

Multimedia Appendix 2 Interview topic list.

Multimedia Appendix 3 Codetree, displaying all applied codes, grouped in colours around every single aspect of quality ofcare.

## Abbreviations

CJG: Centre for Youth and Family
COREQ: Consolidated Criteria for Reporting Qualitative Studies
PAEHR: patient-accessible electronic health record
WHO: World Health Organization

## Data Availability

As interview transcripts contain sensitive information, these will not be published in a separate data set.
