# Supplementary material for: The Effect of Using a Client-Accessible Health Record on Perceived Quality of Care: Interview Study Among Parents and Adolescents
Source: J Particip Med. 2024 Apr 23;16:e50092. doi: 10.2196/50092 (PMC11077414; doi:10.2196/50092)
Supplement: Multimedia Appendix 1 [file jopm_v16i1e50092_app1.docx]

Multimedia Appendix 1: Consolidated criteria for reporting qualitative studies (COREQ): 32-item checklist**^[[1]](#endnote-1)^**

| No | Item | Guide questions/description | | Remarks | Section, subsection |
| --- | --- | --- | --- | --- | --- |
| **Domain 1: Research team and reflexivity** | | | | | |
| *Personal Characteristics* | | | | | |
| 1. | Interviewer/facilitator | Which author/s conducted the interview or focus group? | | Main author JB led all interviews and focus groups  CdM participated in 3 focus groups and 3 interviews as observer and notetaker | Methods, data collection |
| 2. | Credentials | What were the researcher’s credentials? *E.g. PhD, MD* | | JB: MD, public health.  CdM: MSc, Health Biology and Education. | Appendix 1 |
| 3. | Occupation | What was their occupation at the time of the study? | | JB: medical policy advisor in preventive child health care, PhD-candidate  CdM: postgraduate research assistant | Methods, data collection. |
| 4. | Gender | Was the researcher male or female? | | JB: female  CdM: female | Methods, data collection, Appendix 1 |
| 5. | Experience and training | What experience or training did the researcher have? | | JB has several years of experience in qualitative research.  CdM followed workshops about qualitative research and analyzing data with Atlas.TI | Methods, research team and reflexivity |
| *Relationship with participants* | | | | | |
| 6. | Relationship established | Was a relationship established prior to study commencement? | | Both JB and CdM had no professional-client relationship with any of the participants. | Methods, research team and reflexivity |
| 7. | Participant knowledge of the interviewer | What did the participants know about the researcher? *e.g. personal goals, reasons for doing the research* | | At the start of each interview, JB explained the purpose of developing EPR-Youth, her drive to do this research and the purpose of this interview. | Methods, research team and reflexivity |
| 8. | Interviewer characteristics | What characteristics were reported about the interviewer/facilitator? *e.g. Bias, assumptions, reasons and interests in the research topic* | | As a policy advisor, JB was very well informed about the functionalities of EPR-Youth and its patient portal, which was helpful when participants were not yet acquainted with the system. | Methods, research team and reflexivity; discussion. |
| **Domain 2: study design** | | | | | |
| *Theoretical framework* | | | | | |
| 9. | Methodological orientation and theory | What methodological orientation was stated to underpin the study? *e.g. grounded theory, discourse analysis, ethnography, phenomenology, content analysis.* | | A thematic analysis was conducted from a phenomenological perspective, with an aim to determine what using EPR-Youth meant to clients in terms of quality of care. | Methods, research design |
| *Participant selection* | | | | | |
| 10. | Sampling | How were participants selected? *e.g. purposive, convenience, consecutive, snowball* | | Purposive sampling, aiming for a presentation of both sexes, parents and adolescents, different educational levels, both native and migrant background, coming from all participating municipalities and visitors of both preventive health care and youth and social care. | Methods, study population and inclusion. |
| 11. | Method of approach | How were participants approached? *e.g. face-to-face, telephone, mail, email* | | Face-to-face invitation by a professional, followed by phone/email from one of the researchers to make appointment. | Methods, study population and inclusion. |
| 12. | Sample size | How many participants were in the study? | | 20 participants were included, 13 parents and 7 adolescents | Results, general characteristics. Table 1: ‘characteristics participants client interviews’ |
| 13. | Non-participation | How many people refused to participate or dropped out? Reasons? | | 3 adolescents dropped out because of agenda mismatches (initially, 10 adolescents were recruited) | Results, general characteristics |
| *Setting* | | | | | |
| 14. | Setting of data collection | Where was the data collected? *e.g. home, clinic, workplace* | | Where feasible, small group at CJG-Office. If not, location of client’s choice. Three interviews were conducted at the CJG office (one single, one double, and one triple interview), and five interviews were conducted at a client’s home address (two single, one double and two triple interviews). Four individual interviews were conducted online. | Methods, study population and inclusion. |
| 15. | Presence of non-participants | Was anyone else present besides the participants and researchers? | | No | n/a |
| 16. | Description of sample | What are the important characteristics of the sample? e*.g. demographic data, date* | | Demographics (sex, adult/adolescent, educational level, native country and municipality), interview setting and acquaintance with client portal have been represented in table 2 | Results, table 2 |
| *Data collection* | | | | | |
| 17. | Interview guide | Were questions, prompts, guides provided by the authors? Was it pilot tested? | | An interview guide was written, based on the six pillars of quality of care, defined by the Institute of Medicine (1999). The interview guide was written after a group session with professionals, discussing how using EPR-Youth could affect the way parents and adolescents experienced quality of care in each of these six domains. | Methods, data collection |
| 18. | Repeat interviews | Were repeat interviews carried out? If yes, how many? | | No | n/a |
| 19. | Audio/visual recording | Did the research use audio or visual recording to collect the data? | | All interviews were audio recorded. The online meetings were video recorded as well. | Methods, data collection |
| 20. | Field notes | Were field notes made during and/or after the interview or focus group? | | No | n/a |
| 21. | Duration | What was the duration of the interviews or focus group? | | Individual interviews ranged from 30 to 60 minutes, while group interviews took approximately 90 minutes. | Methods, data collection |
| 22. | Data saturation | Was data saturation discussed? | | Yes | Methods, data analysis |
| 23. | Transcripts returned | Were transcripts returned to participants for comment and/or correction? | | All transcripts were returned to participants for a member content check. | Methods, data analysis |
| **Domain 3: analysis and findings** | | | | | |
| *Data analysis* | | | | | |
| 24. | Number of data coders | | How many data coders coded the data? | Two authors (JB and CdM) | Methods, data analysis |
| 25. | Description of the coding tree | | Did authors provide a description of the coding tree? | Yes | Multimedia Appendix 2, Methods, interview outcomes. |
| 26. | Derivation of themes | | Were themes identified in advance or derived from the data? | Themes were identified in advance. The major themes were derived from the IOM definition of Quality of Care. | Methods, data analysis |
| 27. | Software | | What software, if applicable, was used to manage the data? | Atlas.TI, version 9, was used. | Methods, data analysis |
| 28. | Participant checking | | Did participants provide feedback on the findings? | No comments or corrections were returned. Eight participants responded that they had no comments. | Results, interview outcomes |
| *Reporting* | | | | | |
| 29. | Quotations presented | | Were participant quotations presented to illustrate the themes / findings? Was each  quotation identified? *e.g., participant numbe*r | Yes, identified by gender, parent/adolescent, number of children (parents) or age (adolescent), and respondent number. The respondent number is indicated as R followed by the number of the interview, and if more participants were present at the interview a decimal to indicate this specific participant. e.g., R7.3 is the third participant in interview nr 7. | Results, interview outcomes |
| 30. | Data and findings consistent | | Was there consistency between the data presented and the findings? | Yes | Results, interview outcomes |
| 31. | Clarity of major themes | | Were major themes clearly presented in the findings? | Yes | Results, interview outcomes |
| 32. | Clarity of minor themes | | Is there a description of diverse cases or discussion of minor themes? | One major theme was divided in two subthemes. In the discussion, an additional theme ‘integrated care’ was proposed. | Results, interview outcomes; discussion. |

1. Allison Tong, Peter Sainsbury, Jonathan Craig, Consolidated criteria for reporting qualitative research (COREQ): a 32-item checklist for interviews and focus groups, *International Journal for Quality in Health Care*, Volume 19, Issue 6, December 2007, Pages 349–357, DOI 10.1093/intqhc/mzm042 [↑](#endnote-ref-1)
