# Supplementary material for: The Effect of Using a Client-Accessible Health Record on Perceived Quality of Care: Interview Study Among Parents and Adolescents
Source: J Particip Med. 2024 Apr 23;16:e50092. doi: 10.2196/50092 (PMC11077414; doi:10.2196/50092)
Supplement: Multimedia Appendix 2 [file jopm_v16i1e50092_app2.docx]

## Multimedia Appendix 2: Interview topic guide

This topic list guides the interviews with parents and adolescents about their perceptions of quality of care in relation to the use of EPR-Youth (Jeugddossier)

Each interview starts with a short introduction:

- Introduction round interviewers and participants.
- What is today’s topic: what is EPR-Youth and what is its purpose?
- Explanation about research topic and methods, about data management and privacy; request permission to record the meeting; explanation of member check afterward.
- Two general questions:
  - Are you acquainted with EPR-Youth already and do you use it?
    - If participants are not acquainted with EPR-Youth yet, continue with a 10-minute demo of the client portal.
  - What are your experiences? Do you read, ask questions, plan and manage your appointments, do you experience that you can decide who has access to your (child’s) record? If so, what does that mean to you?
    - If participants were not acquainted with EPR-Youth yet, discuss their first impressions with them.
- Continue the conversation, approaching the different **domains of quality of care**:
  - ***Equity***
    - Did you manage to access the client portal? How easy was it? Did you need any help?
    - After accessing the client portal: Do you understand what you read? Can you find everything that you are looking for?
    - If you have never accessed the client portal before: did you know about the existence of EPR-Youth? How would you like to be informed?
  - ***Client-centeredness***
    - How does being able to read EPR-Youth affect the collaboration and/or communication between you and CJG-professionals? (key words: transparency, honesty, trust)
    - In dialogue with parents and adolescents we stated that as CJG-professionals we consider it important to take our clients seriously. Does EPR-Youth contribute to that? How does it affect the sense of equality?
    - If you ask questions, we would like to collaborate with you to find an answer that matches your needs. We consider you the ‘owner’ of your question, therefore you are the one who should choose the solution and who should decide when you have reached your goals. Does EPR-Youth contribute to that ownership of questions, solutions, and goals? Does it change your sense of involvement?
  - ***Safety***
    - How do you feel about the security of your data in EPR-Youth? Has that feeling changed in comparison with the former health records? (each organization using their own health record, no client-access)
    - Do you have any concerns about things going wrong with your data and what would that be?
    - Have you ever discovered flaws in our reports when reading your (child’s) record? If so, what did you do, with what positive/negative consequences?
  - ***Effectivity and Efficiency***
    - How did reading reports influence your view on our professional’s expertise?
    - We work with the ‘1-family-1-plan’ principle: preventive child healthcare professionals and youth care workers report in the same record although they work in different organizations. They can read each other’s reports, which would help them to work more effectively and efficiently. Did you notice any differences because of their shared access?
  - ***Timeliness***
    - You have 24/7 access to your client portal and therefore you can ask questions and manage appointments at any time you like. Does that increase the speed of getting answers to your questions and do you get appointments faster?
    - How do you value the opportunity to ask questions 24/7 in the portal?
    - How do you value the opportunity to check and manage your appointments 24/7? This opportunity only exists for parents visiting preventive health care for preschool children. If you visited preventive healthcare for older children or youth care, would you value the opportunity to check and manage appointments?
