# Supplementary material for: The Effect of Using a Client-Accessible Health Record on Perceived Quality of Care: Interview Study Among Parents and Adolescents
Source: J Particip Med. 2024 Apr 23;16:e50092. doi: 10.2196/50092 (PMC11077414; doi:10.2196/50092)
Supplement: Multimedia Appendix 3 [file jopm_v16i1e50092_app3.pdf]

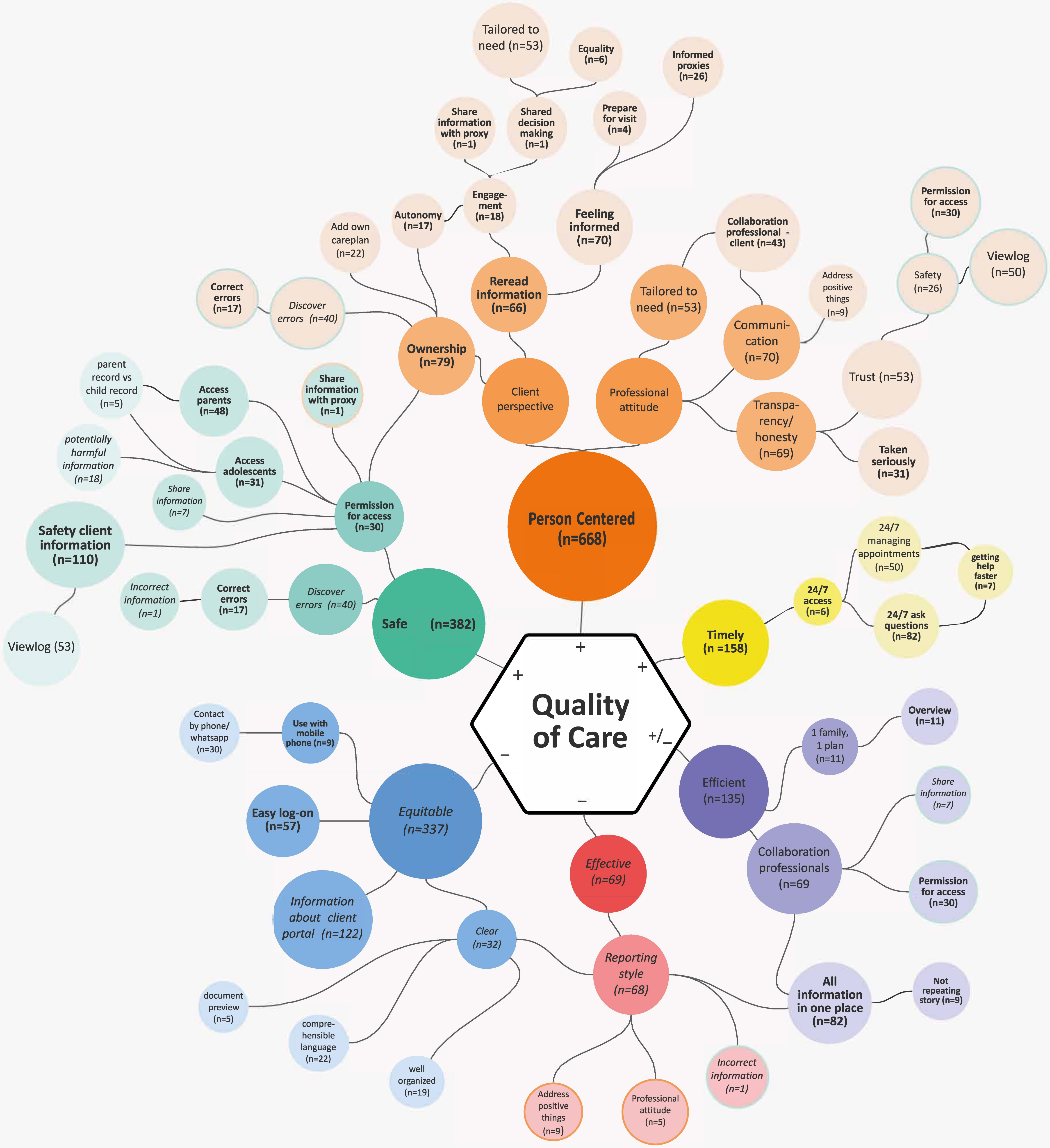

Multimedia Appendix 3: Codetree for perceptions of quality of care, reported by parents and adolescents using Iuvenelis. The main themes are derived from the IOM framework for quality of care, each theme in a different color: Person-centered (orange), Safe (green), Timely (yellow), Efficient (purple), Effective (red), Equitable (blue). Codes with a higher density are represented in a larger circle and larger font. Cursivated codes indicate a mainly negative association with quality of care, bolded codes indicate a mainly positive association with quality of care, codes in normal font are associated both positively and negatively with quality of care. Some codes are connected between themes. If possible, the connection is made with a line from one theme to another. When themes are on opposite sides of the codetree, a code is added to the other theme in the color of that theme and outlined in the color of the original theme. E.g. 'incorrect information' is a code under the green theme 'Safe', and is added under 'Effective' in red with a green outline.
